# Prediction of antimicrobial resistance in clinical *Campylobacter jejuni* isolates from whole-genome sequencing data

**DOI:** 10.1007/s10096-020-04043-y

**Published:** 2020-09-24

**Authors:** Louise Gade Dahl, Katrine Grimstrup Joensen, Mark Thomas Østerlund, Kristoffer Kiil, Eva Møller Nielsen

**Affiliations:** Department of Bacteria, Parasites & Fungi, Statens Serum Institut, Copenhagen, Denmark

**Keywords:** *Campylobacter jejuni*, Antimicrobial resistance, Whole-genome sequencing, Public health, AMR surveillance

## Abstract

*Campylobacter jejuni* is recognised as the leading cause of bacterial gastroenteritis in industrialised countries. Although the majority of *Campylobacter* infections are self-limiting, antimicrobial treatment is necessary in severe cases. Therefore, the development of antimicrobial resistance (AMR) in *Campylobacter* is a growing public health challenge and surveillance of AMR is important for bacterial disease control. The aim of this study was to predict antimicrobial resistance in *C. jejuni* from whole-genome sequencing data. A total of 516 clinical *C. jejuni* isolates collected between 2014 and 2017 were subjected to WGS. Resistance phenotypes were determined by standard broth dilution, categorising isolates as either susceptible or resistant based on epidemiological cutoffs for six antimicrobials: ciprofloxacin, nalidixic acid, erythromycin, gentamicin, streptomycin, and tetracycline. Resistance genotypes were identified using an in-house database containing reference genes with known point mutations and the presence of resistance genes was determined using the ResFinder database and four bioinformatical methods (modified KMA, ABRicate, ARIBA, and ResFinder Batch Upload). We identified seven resistance genes including *tet(O)*, *tet(O/32/O)*, *ant(6)-Ia*, *aph(2″)-If*, *blaOXA*, *aph(3′)-III*, and *cat* as well as mutations in three genes: *gyrA*, *23S rRNA*, and *rpsL*. There was a high correlation between phenotypic resistance and the presence of known resistance genes and/or point mutations. A correlation above 98% was seen for all antimicrobials except streptomycin with a correlation of 92%. In conclusion, we found that WGS can predict antimicrobial resistance with a high degree of accuracy and have the potential to be a powerful tool for AMR surveillance.

**Electronic supplementary material:**

The online version of this article (10.1007/s10096-020-04043-y) contains supplementary material, which is available to authorized users.

## Introduction

*Campylobacter* is a foodborne bacterial pathogen, which is able to cause gastroenteritis in humans and is found in the environment where it is widely distributed in the gastrointestinal tract of most warm-blooded animals [1]. Foodborne transmission is believed to be the main route of human infection [2, 3]. In 2018, *Campylobacter* continued to be the most common foodborne pathogen causing infections in Denmark with more than 4500 registered cases; most of these were caused by *C. jejuni* (85–95%) [4]. Clinical symptoms during infection often include diarrhoea, fever, abdominal pain, nausea, headache, and/or vomiting [5, 6] and in some cases result in Guillain-Barré syndrome (GBS) or reactive arthritis [7, 8]. Most patients recover without any antimicrobial treatment, whereas more severe and immunocompromised cases may need antibiotic treatment. Due to the extensive use of antimicrobials worldwide, within the production of food animals and human medicine, antimicrobial resistance (AMR) against clinically relevant antibiotics is emerging in C*ampylobacter* spp. presenting a serious health concern [2, 9, 10]. In Denmark, AMR surveillance in *Campylobacter jejuni* has been in place since 1996 based on phenotypic susceptibility testing against ciprofloxacin (CIP), nalidixic acid (NAL), erythromycin (ERY), gentamicin (GEN), streptomycin (STR), and tetracycline (TET). The data on clinical isolates is reported as part of The Danish Integrated Antimicrobial Resistance Monitoring and Research Programme (DANMAP) (https://www.danmap.org/).

*Campylobacter* has developed several mechanisms for antibiotic resistance including point mutations, acquisition of resistance genes, and efflux systems [11–13]. Resistance towards fluoroquinolones (FQs) in *Campylobacter* is mainly caused by point mutations in the DNA gyrase A (GyrA) region at multiple positions [10, 14]. The mechanisms involved in erythromycin resistance include ribosomal target modification and efflux of the antibiotic from the cell and this resistance is often associated with point mutation in domain V of the 23S rRNA, which is the target of macrolides, or the ribosomal proteins L4 and L22 [10, 15]. *Campylobacter* harbour three copies of the 23S rRNA gene, but only two copies need to be affected by point mutations to result in erythromycin resistance [16, 17]. Streptomycin resistance in *Campylobacter* is associated with substitution within the ribosomal RpsL protein or by expression of an aminoglycoside-modifying enzyme (ANT(6)-I) [18, 19]. Resistance towards gentamicin and tetracycline is in general conferred by the acquisition of resistance-associated genes. Resistance to tetracycline in *Campylobacter* is mainly conferred by the ribosomal protection protein *tet(O)*, a plasmid-encoded gene, whereas the resistance gene *aph(2″)-If* often is associated with gentamicin resistance [20–22].

The whole-genome sequencing (WGS) technology provides complete genomic DNA sequence of a bacterium, and WGS has proved to be an effective surveillance tool for foodborne pathogens [23, 24]. This method provides a large amount of information generated from a single assay [25] and allows multiple tests to be performed in silico simultaneously, whereas phenotypic susceptibility tests can be time-consuming, labour intensive, and are highly dependent on standardised laboratory procedures. Several studies have demonstrated that WGS can be used to identify resistant genotypes for different foodborne pathogens [23, 24, 26]. The aim of this study was to evaluate the concordance between WGS-based AMR prediction and the phenotypes identified from conventional antimicrobial susceptibility in a set of clinical *C. jejuni* isolates. The focus of the evaluation is to allow the replacement of phenotypic testing with a WGS-based surveillance of AMR in *Campylobacter*.

## Materials and methods

### Bacterial isolates

Clinical *C. jejuni* isolates representing common and less common resistance profiles were included as the study population. In total, 516 clinical *C. jejuni* isolates from Danish patients were analysed. The isolates were submitted to SSI from 2014 to 2017. Of the submitted isolates, 422 were from domestically acquired infections, 84 were travel related, and 10 isolates had no available travel information. At SSI, the isolates were subjected to MALDI-TOFF for species confirmation, and antimicrobial susceptibility was tested as described below.

### Antimicrobial susceptibility testing

Isolates were grown on modified charcoal cefoperazone deoxycholate agar (mCCDA) plates. After overnight incubation, one colony was selected and inoculated on blood agar plates. This culture was used for AMR testing and DNA purification for WGS. Incubation was performed in 18 to 24 h under microaerophilic conditions (85% nitrogen, 10% carbon dioxide, 5% oxygen) at 41 °C.

Minimum inhibitory concentration (MIC) values were determined against six antimicrobials: CIP, NAL, ERY, GEN, STR, and TET using the Trek Sensititre Broth Microdilution setup using the EUCAMP plate design (ThermoFisher Scientific) that includes the test ranges recommended by ECDC [27]. MIC values were interpreted as susceptible (wild-type) or resistant (non-wild-type) using epidemiological cutoff (ECOFF) defined by EUCAST: CIP (> 0.5 μg/ml), NAL (> 16 μg/ml), ERY (> 4 μg/ml), GEN (> 2 μg/ml), STR (> 4 μg/ml), and TET (> 1 μg/ml) [27].

### Whole-genome sequencing

Genomic DNA was purified from pure cultures using QIAGEN DNeasy Blood & Tissue Kit (https://www.qiagen.com). The DNA concentration was measured using an Invitrogen Qubit Fluorometer (ThermoFisher Scientific). After adjustment of the concentration, the isolates were sequenced using Illumina sequencing technology (https://www.illumina.com) on either Miseq or Nextseq sequencing machines using the Nextera XT Library preparation protocol for paired-end reads of 150 bp or 250 bp. The sequence data was evaluated by an in-house quality-control pipeline (https://github.com/ssi-dk/bifrost) to check for contaminations (maximum 5% of other genus allowed), correct species identification, and read depth and genome size. Sequences representing *C. jejuni* isolates with a genome size outside the range of 1.6–1.9 mbp were excluded as well as if the number of contigs exceeded 500. Coverage depth of the included genomes ranged from 32 to 506 (median 111). Data analysis was performed in BioNumerics version 7.6 (Applied Maths) where 7 locus MLST was assigned and phylogenetic minimum spanning trees were generated.

### AMR prediction

The presence of acquired resistance genes was determined by four approaches: two based on prediction from methods using de novo assemblies (ResFinder Batch Upload (web tool) [28] and ARIBA [29]) and two methods using reference mapping (KMA [30] and ABRicate, available at https://github.com/tseemann/abricate). All methods utilised the ResFinder gene database for detection and for all four approaches, hits were only considered for gene lengths ≥ 90% and identities of ≥ 60%. When alleles were not represented in the database (i.e., novel alleles of a present resistance gene), reference mapping by the use of KMA splits the mapping between two or more references in the database. To alleviate this in our setup, an additional step was added to perform another mapping against the reference gene with the highest coverage in order to obtain a result with a full-length gene variant (modified KMA; https://github.com/ssi-dk/modified_kma).

For detection of point mutations conferring antimicrobial resistance, a database containing reference genes (*gyrA*, *rplD*, *rplV*, *rpsL*, and *23S*) and associated point mutations known from the literature to be coupled with antimicrobial resistance in *C. jejuni* was constructed from *C. jejuni* strain NCTC 11168 (Table 1). Point mutations were detected using a local wrapper (https://github.com/ssi-dk/punktreskma) for KMA [30]. For *23S* rRNA, where multiple gene copies (3) are present in the genome, the detection of a certain nucleotide at a certain position was done by considering the particular depth for each nucleotide, specifically by assuming a uniform distribution of reads over gene copies. Thus, the count of each nucleotide was assumed to be the fraction of reads it was observed on. Of note, this method does not consider the co-occurrence of point mutations on single reads.

**Table 1:** Chromosomal point mutations resulting in AMR in *C. jejuni*

Antibiotic class	Gene(s)	Point mutation			Reference(s)
		Verified^a^	Not verified^b^	Not found^c^	
(Fluoro)quinolone	*gyrA*	T86I, D90N, T86A	P104S	A70T, D85Y, T86K, T86V, D90A, D90T, D90Y	[31–35]
Macrolide (ERY)	*rplD* *rplV*		D72N, A103V	G74D, G67V, A71D, R72I G86E, A88E, A103C A2074G, A2074C	[36] [34–37] [31, 36]
	*23S*	A2075G, A2074T			
Aminoglycoside (STR)	*rpsL*	K43R, K88R		K88E, K88Q	[38, 39]

^a^Verified: point mutations were found in the study population and these correlated with the relevant phenotype

^b^Not verified: point mutations found in study population but not possible to connect to phenotype

^c^Not found in data: point mutations not found within study population

In addition, we included in the database the point mutations G86A and C696T in the promotor region (P_cmeABC_) of the efflux system cmeABC. These point mutations have been hypothesised to increase the level of resistance towards CIP /NAL, ERY, and TET [31, 40, 41].

For each isolate, phenotypic susceptibility to a given antimicrobial agent was compared with the presence of acquired resistance genes and resistance-associated point mutations found by WGS. The correlation between resistant phenotypes and genotypes was calculated by dividing the number of isolates considered resistant based on their genotype by the number of isolates exhibiting resistant phenotypes.

Susceptibility against the six antimicrobials was retested for 62 isolates and new sequence data was obtained for 47 of these to consolidate observed discrepancies between phenotype and predicted genotype.

## Results

### In vitro antimicrobial susceptibility testing

Phenotypic susceptibility testing showed that 277 of the 516 clinical isolates (54%) were sensitive to all six antimicrobials (Table 2). Among isolates from domestically acquired infections, 61% (258/422) were fully sensitive compared with 18% of isolates from travel-related infections (15/84).

**Table 2:** Phenotypic resistance profiles of the 516 clinical isolates based on the test panel, including CIP, NAL, ERY, GEN, STR, and TET

Resistance profile	Domestic	Travel	N/A	Total
Fully sensitive	258	15	4	277
CIP-NAL	92	21	1	114
CIP-NAL-TET	49	34	4	87
TET	14	1		15
CIP-NAL-ERY-TET	3	4		7
CIP-NAL-STR-TET	1	3	1	5
STR	3			3
CIP-NAL-ERY-STR	2			2
CIP-NAL-ERY-GEN-TET		1		1
CIP-NAL-ERY-STR-TET		1		1
CIP-NAL-GEN-TET		1		1
CIP-STR-TET		1		1
CIP-TET		1		1
NAL-TET		1		1
Total	422	84	10	516

Thirteen different resistance profiles were identified and the most frequent was CIP/NAL (114/516; 22%), followed by CIP/NAL-TET (87/516; 17%) (Table 2). No isolates were resistant to all six antimicrobials.

**Fig. 1:**
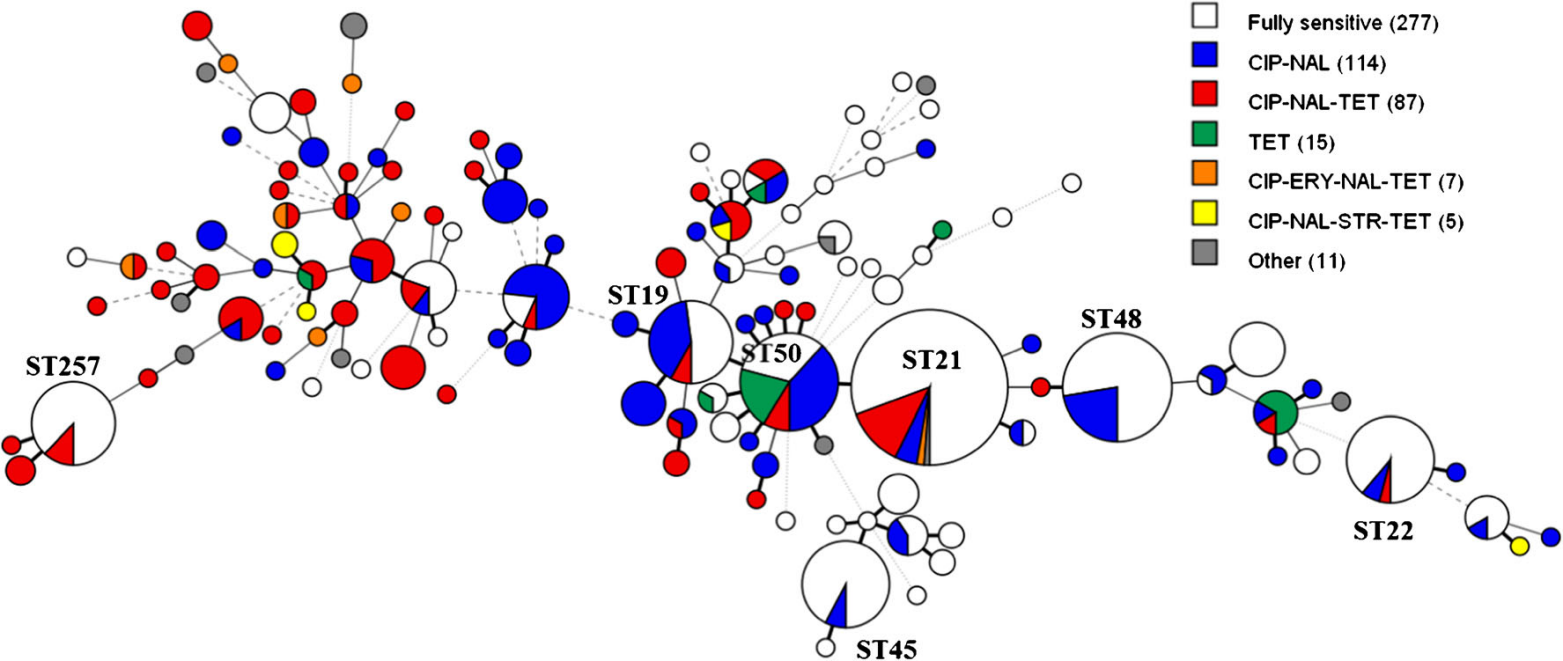
Minimum spanning three generated from 7 locus MLST showing phenotypic resistance profiles of the 516 *C. jejuni* isolates. Each node represents an ST and the size of the node correlates to the number of isolates. Isolates resistant to one or more of the six antimicrobials are coloured. Grey nodes (other) represent less common resistance profiles seen for three or less isolates. Isolates susceptible to all six antimicrobials are uncoloured. Numbers in the parentheses indicate number of isolates with the respective resistance profile

The distribution of phenotypic resistance in the study population is illustrated in Fig. 1. All phenotypic resistance profiles were widely represented in the population and the 43% quinolone-resistant isolates were found in most major sequence types (ST).

### Concordance between phenotypic and genotypic AMR

In the study population, known resistance-associated point mutations were observed for *gyrA*, *23S*, and *rpsL* mediating resistance to CIP/NAL, ERY, and STR, respectively. Some resistance-associated point mutations were not found in the study population (Table 1) and therefore, it was not possible to verify these. After retesting, it was found that most deviations were due to an error in the phenotypic resistance profile and only seven isolates ended up with a new sequence after WGS rerun (possible mix-up of isolates). An overview of the detected resistance-associated point mutations and acquired resistance genes is shown in Table 3.

**Table 3:** Correlation of resistance phenotype and predicted genotype for 516 Danish clinical *C. jejuni* isolates. The result of identified resistance genes is found based on the modified KMA

Drug class	Tested drugs	No. of isolates with R or S phenotype	Detected genes and mutations	Correlation between phenotype and genotype (%)
(Fluoro)quinolone	CIP/NAL	R (*n* = 218))	*gyrA* T86I (*n* = 199) *gyrA* T86I + P104S (*n* = 13) *gyrA* T86I + D90N (*n* = 1) *gyrA* D90N (*n* = 1) None (*n* = 4)	98 (214/218)
	NAL	R (*n* = 1		
			*gyrA* T86A (*n* = 1)	
	CIP	R (*n* = 2)	*gyrA* T86I (*n* = 2)	
		S (*n* = 295)	None (*n* = 295)^a^	100 (295/295)
Macrolide	ERY	R (*n* = 11)	*23S* rRNA A2075G (*n* = 8)^b^ *23S* rRNA A2074T (*n* = 3)	100 (11/11)
		S (*n* = 505)	None (*n* = 505)^c^	100 (505/505)
Aminoglycoside	GEN	R (*n* = 2)	*aph(2″)-If* (*n* = 2)	100 (2/2)
		S (*n* = 514)	None (*n* = 514)	100 (514/514)
Aminoglycoside	STR	R (*n* = 12)	*rpsL* K43R (*n* = 4) *rpsL* K88R (*n* = 1) *ant(6)-Ia* (*n* = 6) None (*n* = 1)	92 (11/12)
		S (*n* = 504)	None (*n* = 504)	100 (504/504)
Tetracycline	TET	R (*n* = 120)	*tet(O)* (*n* = 90) *tet(O/32/O)* (*n* = 27) None (*n* = 3)^d^	98 (117/120)
		S (*n* = 396)	None (*n* = 385) Non-functional *tet(O)* (*n* = 11)	100 (396/396)

Markers not verified in this study: ^a^*gyrA* P104S (*n* = 1); ^b^additionally A103V in *rplV* (*n* = 3); ^c^A103V in *rplV* (*n* = 65) and D72N in *rplV* (*n* = 1); ^d^*tet(O)* was identified in a phenotypic TET resistant isolate with a gene length just below the cutoff (89.06%)

The most frequently observed point mutation was the *gyrA* T86I, which was detected in 215/221 (97%) of the quinolone-resistant isolates. In 13 of these isolates, the *gyrA* P104S point mutation was also present. However, the importance of P104S in relation to CIP/NAL resistance could not be verified in this study as P104S was also found in a CIP/NAL sensitive isolate (MIC_CIP_ = 0.25 μg/ml, MIC_NAL_ = 8 μg/ml). We only found one CIP/NAL resistant isolate harbouring the *gyrA* D90N mutation.

Three isolates were phenotypically resistant to just one of the two quinolones. Two CIP-resistant isolates harboured *gyrA* T86I and showed high-level resistance towards CIP (MIC_CIP_ = 16 μg/ml) while being well below the breakpoint for NAL (MIC_NAL_ = 2 μg/ml and 4 μg/ml). One NAL-resistant isolate harboured the *gyrA* T86A mutation and showed high-level resistance towards NAL (MIC_NAL_ = 64 μg/ml) while being just below the breakpoint for CIP (MIC_CIP_ = 0.5 μg/ml). Discrepancies between the phenotype and genotypic prediction were observed for four isolates. These isolates showed a resistant phenotype against CIP/NAL but without any *gyrA* point mutation present.

Eleven (2%) isolates were resistant to ERY and all carried resistance-associated point mutations in the *23S* genes; eight harboured the mutation A2075G, while three harboured the A2074T mutation. Most (10/11) isolates had the same point mutation in all three copies of the 23S rRNA gene. One isolate carried a mutation in only two of the three gene copies, while still displaying high-level resistance (MIC_ERY_ > 128 μg/ml).

It was not possible to verify the importance of two point mutations, D72N and A103V, associated with ERY resistance (Table 1). We found that 68 isolates harboured A103V but only three isolates showed phenotypic resistance towards erythromycin (MIC > 128 μg/ml) and these isolates also harboured point mutation in *23S* at position 2075. Furthermore, D72N was also found in an erythromycin sensitive isolate.

Among the 516 isolates, seven different acquired resistance genes were identified. Four of these genes are known to mediate resistance towards antimicrobials represented in the phenotypic panel: TET (*tet(O)* and *tet(O/32/O)*), STR (*ant(6)-Ia*), GEN (*aph(2″)-If*), while genes not included in the test panel also were detected: beta-lactam (*blaOXA*), some aminoglycosides (*aph(3′)-III*), and chloramphenicol (*cat*).

**Fig. 2:**
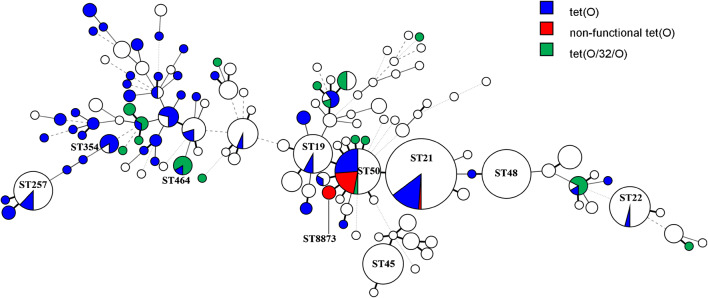
Minimum spanning three generated from MLST showing the distribution of genotypic markers of tetracycline resistance, including the non-functional *tet(O)* gene found in 11 phenotypically susceptible isolates

Phenotypically, 120 (23%) of the isolates were resistant to tetracycline and this was conferred by either *tet(O)* or *tet(O/32/O)*. Furthermore, 11 of the 396 (3%) susceptible isolates carried *tet(O)* (Table 3); however, all of these were found to be a non-functional variant (frameshift mutation). For 117/120 (98%) of the phenotypically resistant isolates, a TET resistance gene was detected by modified KMA: 90 isolates harboured the *tet(O)* gene and 27 the mosaic gene *tet(O/32/O)*. Figure 2 shows that TET resistance occurred widespread in the population. The less common gene, *tet(O/32/O)*, was rare in the most common STs and the non-functional variant of *tet(O)* was only found in isolates of ST21 (*n* = 1), ST50 (*n* = 7), and the closely related ST8873 (*n* = 3).

Only two isolates in the study population were resistant to the aminoglycoside GEN, both with high-level resistance (MIC_GEN_ ≥ 16 μg/ml) and harbouring the resistance gene *aph(2″)-If*.

Twelve (2%) isolates were STR resistant, and 11/12 (92%) possessed either the *ant(6)-Ia* (*n* = 6) or one of the known *rpsL* point mutations (*n* = 5) (Table 3). The resistant isolate (MIC_STR_ = 16 μg/ml) without any of these markers carried the *aph(3′)-III* gene, which is known to confer resistance to other aminoglycosides than STR. This gene was also found in three STR susceptible isolates.

We investigated the presence of the point mutations G86A and C696T in the promotor region of CmeABC (P_cmeABC_) in the study population. G86A was not found in any of the isolates, but the point mutation C696T was identified in 21/516 (4%) isolates. All 21 isolates showed resistance to CIP/NAL, 19 against TET, and two against ERY, where all isolates had a known point mutation and/or acquired resistance gene facilitating the resistant phenotype. Comparing the MIC values for these 21 isolates with the same point mutation and/or resistant gene without C696T showed significantly (*P* < 0.05) higher MIC values to CIP/NAL.

### Evaluating modified KMA, ABRicate, ARIBA, and ResFinder Batch Upload

The detection of resistance genes was performed using four different methods (KMA, ABRicate, ARIBA, and ResFinder Batch Upload). In general, there was a high conformity between the four approaches; however, the mapping-based method modified KMA resulted in more resistance genes being identified and therefore the preferred method to use. The four methods identified four genes relevant to the phenotypic panel (Table 4).

**Table 4:** Overview of identified genes related to the antimicrobial agents in the test panel, based on four different methods (modified KMA ABRicate, ARIBA, ResFinder Batch Upload)

Gene	Mapping		Assembly	
	Modified KMA	ABRicate	ARIBA	ResFinder Batch Upload
*tet(O)*	90^a^	91^b^	92^b^	91
Non-functional *tet(O)*	11	11	11	11
*tet(O/32/O)*	27	3	0	0
*ant(6)-Ia*	6	6	6	6
*aph(2″)-If*	2	2	2	2

^a^*tet(O)* was identified in one isolate with a gene length just below the cutoff (89.06%) using modified KMA and therefore considered not present using this method. *tet(O)* was detected in this isolate by ARIBA and ResFinder Batch Upload

^b^One isolate was found to carry *tet(O)* by ABRicate and ARIBA, but *tet(O/32/O)* by modified KMA

All applied methods were able to identify *aph(2″)-If* in the two GEN-resistant isolates and *ant(6)-Ia* in six streptomycin-resistant isolates with high gene coverage and identity. The *tet(O)* gene was detected in 90 isolates by all methods. In one additional isolate, ResFinder Batch Upload and ARIBA detected *tet(O)*, which was not detected by ABRicate and modified KMA. However, in this isolate, modified KMA identified *tet(O)* with a gene length just below the cutoff (89.06%). For one isolate, the methods found different *tet* genes (all full-length and above 99% identity): KMA detected *tet(O/32/O)* whereas ABRicate and ARIBA found *tet(O)*. It was only the mapping-based methods, KMA and ABRicate, that were able to detect *tet(O/32/O)* with a gene length > 90%; KMA in 27 isolates, and ABRicate in three of these isolates. Additionally, ABRicate and ARIBA identified *tet(O/32/O)* in 16 and 26 resistant isolates, respectively, but with insufficient gene lengths, ranging from 5 to 78%. ResFinder Batch Upload could not identify *tet(O/32/O)* in any isolate with the settings used.

Additional resistance genes, not related to the phenotypic test panel, were also found and included *blaOXA*, *aph(3′)-III* and *cat* (results not shown). Modified KMA and ABRicate identified *blaOXA*, mediating resistance towards beta-lactam, within 458 and 456 isolates, respectively, whereas ARIBA and Resfinder found *blaOXA* in 459 and 429 isolates, respectively. For 420 of these isolates, all methods identified *blaOXA* genes and in the remaining isolates, not all methods identified a variant of the *blaOXA* gene or the gene length of *blaOXA* was < 90%. The *aph(3′)-III* gene was identified by all methods in six isolates. The *cat* gene, mediating chloramphenicol resistance, was found in one isolate by modified KMA, ARIBA, and ResFinder Batch Upload.

## Discussion

Resistance against clinically important antibiotics in *Campylobacter* continues to be a public health concern worldwide. *Campylobacter* is exposed to antibiotics in both animal production and human medicine making development and transmission of AMR possible. To assess the ability of using WGS to identify and predict AMR in *C. jejuni*, a dataset of 516 clinical isolates were analysed by comparing their resistance genotypes to their respective phenotypic resistance profile. The resistance profiles were found based on susceptibility to six antimicrobials all included in the Danish antimicrobial surveillance programme, DANMAP [2]. In this study, we evaluated and optimised the analysis of WGS data to identify—with high confidence—the relevant genetic markers that enable the prediction of the phenotypic resistance against specific antimicrobials.

For the genome-based prediction of AMR, we analysed WGS data by the use of mapping- and assembly-based bioinformatics methods to detect known AMR genes listed in a curated database, ResFinder. For detection of point mutations, we constructed a database containing reference genes and associated point mutations known from the literature. For each of the six antimicrobials tested, there was a concordance between 92 and 100% between the presence of known resistance genes or mutations and MICs above the ECOFF breakpoint. To obtain this high concordance, a number of modifications were made to the raw output from the ResFinder database search as well as the detected point mutations validated before inclusion in the final output presented here.

The four approaches to identify the presence of acquired resistance genes (modified KMA, ABRicate, ARIBA, ResFinder Batch Upload) worked well and with an overall good compliance between the results obtained, however with a few important differences that made us chose a modified KMA as the preferred method. A systematic error was attained when using three of the four methods for the detection of the mosaic gene *tet(O/32/O)* conferring resistance to tetracycline. Although *tet(O/32/O)* is present in the ResFinder database, only the mapping-based KMA method was able to detect this gene in 27 isolates, whereas neither of the assembly-based methods (ARIBA and ResFinder Batch Upload) detected *tet(O/32/O)* with a gene length > 90% in any of the genomes. This would potentially make an under-estimation of the occurrence of TET resistance as this gene was present in 23% of the TET-resistant genomes and it was widely distributed in our *C. jejuni* population. For all approaches, another systematic error was found to be related to frameshift mutations resulting in non-functional genes. We discovered 11 non-functional *tet(O)* genes resulting in a disagreement between phenotype and genotype. For ten of the isolates, the frameshift was due to a nucleotide deletion, whereas for the last isolate, the *tet* gene harboured an extra nucleotide compared with the reference. As a result, 3% of the TET sensitive isolates were wrongly determined as resistant. Such frameshift-related errors can be avoided by an automated frameshift check of the matched open reading frames or by a tblastx search.

Verification of markers responsible for resistance is essential for building a reliable pipeline. In this study, it was not possible to verify the significance of some point mutations described in the literature. This was the case for the ribosomal protein L22 (*rplV*) and the substitution P104S in *gyrA* associated with resistance against ERY and CIP/NAL, respectively. One mutation in L22 (A103V) was equally common in resistant and sensitive isolates, whereas another mutation in L22 (D72N) was only detected in a sensitive isolate. Thus, our findings suggest that D72N and A103V in L22 do not contribute to erythromycin resistance although these substitutions were reported to be the responsible marker for ERY resistance in one and two isolates, respectively [37, 42]. The P104S substitution in *gyrA* has been found previously in clinical isolates [43, 44], but our data could not support the association to fluoroquinolone resistance, as P104S was found either together with T86I in *gyrA* in resistant isolates or in a phenotypically sensitive isolate. These findings, summarised in Table 1, highlight the importance of being cautious when including suggested mechanisms of resistance in a routine analysis of predicting phenotypic resistance.

The most frequent resistance profile observed among the isolates was CIP/NAL resistance. Quinolone resistance in *Campylobacter* is most often due to mutations in the DNA gyrase A gene, *gyrA*, and especially substitutions at Thr86 are associated with high-level resistance to quinolones [32]. The substitution T86I was present in 97% of the quinolone resistant isolates and none of the sensitive isolates in our study. Two isolates carrying the T86I *gyrA* substitution showed high-level resistance to CIP but were fully susceptible to NAL. This phenomenon is described previously [45] and shows that the exact phenotype may not be possible to predict with the current knowledge. Another mutation, the more atypical T86A *gyrA* substitution, was observed in one isolate that — in accordance with previous studies [45–47] — displayed high-level resistance towards NAL, but was susceptible to CIP. Double point mutations resulting in two amino acid substitutions in *gyrA* were also seen. In addition to the common T86I, 13 isolates had the P104S substitution and one isolate had the D90N substitution. The D90N substitution is less common and associated with moderate resistance [31–33]; however, we detected D90N as the only marker of quinolone resistance in one of the CIP/NAL-resistant isolates.

Resistance to erythromycin is commonly mediated by mutations in *23S* rRNA, especially A2075G [36, 48]. *C. coli* is more often resistant to macrolides than *C. jejuni*, and the *ermB* gene has been shown to mediate resistance to erythromycin in *C. coli* isolates of animal origin [49, 50], but recently *ermB* was also identified in a clinical *C. jejuni* isolate [51]. In this study, none of the isolates had the *ermB* gene. All isolates exhibiting phenotypic resistance against ERY had a mutation in the *23S* rRNA genes at position 2074 or 2075, resulting in a 100% concordance between the phenotype and genotype. None of the isolates had any of the described point mutations in the ribosomal protein L4 (*rplD*).

When disregarding detection of the mentioned non-verified point mutations and the non-functional *tet(O)* genes, discrepancies between phenotype and genotypic prediction were seen for eight isolates, four for CIP/NAL, one for STR, and three for TET. In one of the TET-resistant isolates, a *tet* gene was clearly present as determined by the alternative methods. Otherwise, it is possible that the discrepancies were caused by unknown resistance mechanisms, sequence gaps in the genome preventing detection of known resistance genes or point mutations, or due to mixed cultures in the sample.

Based on our findings, we will establish a routine WGS-based analysis pipeline for AMR detection that we believe has a sufficiently high level of performance for replacing the phenotypic surveillance of AMR in clinical isolates of *Campylobacter*. Phenotypic susceptibility testing may still be optimal for clinical purposes as the WGS approach cannot deliver an MIC value. However, a sequence-based approach predicting MIC values from markers influencing the MICs may be clinically relevant and could be implemented in the database in the future. In our selected *C. jejuni* population, we only detected a limited number of genes and mutations that seem to be relevant for predicting the AMR profile. Thus, future routine analysis will only include the verified mutations and the acquired genes in the curated public databases with focus on avoiding the systematic errors encountered in this study, e.g. non-functional genes and detection of the mosaic *tet* gene. The pipeline should be maintained continuously. We were only able to detect a minority of the many reported point mutations, and therefore, it is relevant to include all potentially important mutations in an additional analysis and evaluate these by phenotypic testing of the isolates. Likewise, new genes or variants of genes may be added to the curated databases, and the performance of our detection pipeline should be evaluated for these. In addition, a subset of the isolates should be tested phenotypically to be able to detect new mechanisms, non-functional genes, and generally perform quality assurance on the analysis pipeline. This further evaluation and development can be done on small selected strain collections at regular intervals.

## Electronic supplementary material

ESM 1 (XLSX 155 kb)

## Data Availability

Sequences are available at ENA, project numbers PRJEB40238 and PRJEB31119. The full antimicrobial resistance data and the individual accession numbers are available in the supplementary file.
